# Exploring Genetic Diversity in a Temperate Bamboo: A Study Using SSR Markers

**DOI:** 10.3390/plants15142240

**Published:** 2026-07-22

**Authors:** Khin Nyein Chan, Janka Bedő, Kitti Andrea Tóth-Lencsés, András Neményi, Csilla Mihályfi, Szilvia Kisvarga, László Orlóci, Anikó Veres

**Affiliations:** 1 Institute of Genetics and Biotechnology, Hungarian University of Agriculture and Life Sciences (MATE), 2100 Gödöllő, Hungary; channyeinkhin1@gmail.com (K.N.C.); bedo.janka@uni-mate.hu (J.B.); mihalyfi.csilla.rita@phd.uni-mate.hu (C.M.); veres.aniko@uni-mate.hu (A.V.); 2 Institute of Landscape Architecture and Garden Art, Hungarian University of Agriculture and Life Sciences (MATE), 2100 Gödöllő, Hungary; toth-lencses.andrea.kitti@uni-mate.hu (K.A.T.-L.); kisvarga.szilvia@uni-mate.hu (S.K.); orloci.laszlo@uni-mate.hu (L.O.)

**Keywords:** *Phyllostachys*, genetic diversity, SSR molecular markers, private allele

## Abstract

Molecular markers have been employed for decades to assess genetic diversity in the *Phyllostachys* genus. However, a more comprehensive understanding of genetic variation among *Phyllostachys* species is still needed to carry out accurate germplasm identification, clarify genetic relationships among taxa, and support the conservation and management of bamboo genetic resources. High genetic diversity within bamboo populations enhances their resilience and adaptability to environmental changes. In this study, we evaluated genetic variation across 96 genotypes representing 44 *Phyllostachys* species using eight simple sequence repeat markers. These markers detected 47 alleles in total, ranging from 3 to 11 per locus. A private allele (201 bp) was identified in *Phyllostachys edulis* and its associated forms using the PBM019 marker. Cluster analysis grouped the samples into three clusters: 22 genotypes in Cluster I, 54 in Cluster II, and 21 in Cluster III. Principal coordinate analysis (PCoA) corroborated these clusters, revealing interspecific genetic divergence alongside high intraspecific and intra-form similarity, except in *Phyllostachys arcana* where two related forms exhibited clear genetic differentiation and clustered separately in both analyses. The PBM021 marker showed the highest polymorphism, amplifying eight alleles in select genotypes (*Phyllostachys angusta*, *P. mannii*, *P. bissetii*, *P. heteroclada*). Overall, this research provides insights into genetic diversity and relationships among 96 bamboo genotypes.

## 1. Introduction

Bamboos (Poaceae: Bambusoideae) comprise more than 1600 species distributed primarily throughout Asia, with additional native and introduced species occurring in America and Europe. They are among the most valuable non-timber forest resources because of their ecological, economic, and cultural importance. Bamboo species are widely used in construction, furniture production, paper manufacturing, food production, bioenergy, and ornamental horticulture [1]. Although bamboos are not indigenous to Europe, numerous species have been introduced into several countries, including Belgium, Germany, France, the Netherlands, Spain, and Italy [2]. Commercial plantations have been established in selected European regions, particularly for edible shoot production, while many species are increasingly cultivated as ornamentals. Among temperate bamboos, the genus *Phyllostachys* is one of the most widely cultivated and taxonomically diverse genera, comprising approximately 69 species, six varieties, and 45 forms [3]. Preserving genetic diversity within *Phyllostachys* is essential because it underpins the adaptive potential of populations, enhances resilience to environmental change, and provides valuable genetic resources for breeding and conservation [4]. However, climate change, habitat degradation, and increasing anthropogenic pressure threaten bamboo genetic diversity by reducing natural populations and potentially eroding unique alleles [5]. Consequently, the characterization and conservation of bamboo germplasm have become increasingly important.

Molecular markers have become indispensable tools for assessing genetic diversity, population structure, and evolutionary relationships in plants. Various marker systems, including random amplified polymorphic DNA (RAPD), amplified fragment length polymorphism (AFLP), inter-simple sequence repeats (ISSR), single nucleotide polymorphisms (SNPs), and simple sequence repeats (SSRs), have been widely employed in plant genetic studies. Among these, SSR markers are particularly valuable because they are abundant throughout plant genomes, highly polymorphic, codominantly inherited, reproducible, and transferable among related taxa [6,7,8,9,10]. These characteristics enable SSRs to provide reliable estimates of genetic diversity and differentiation within and among populations, making them suitable for germplasm characterization, phylogenetic analyses, conservation genetics, and breeding applications [11,12,13,14,15,16,17,18,19,20,21,22,23]. Accordingly, SSR markers have been successfully applied to investigate genetic diversity in numerous bamboo species [24,25,26,27,28,29,30,31,32,33,34,35].

Despite the widespread use of SSR markers in bamboo research, most previous studies have focused on a limited number of species or populations, and comprehensive comparative analyses across multiple *Phyllostachys* species remain relatively scarce. Furthermore, although many bamboo species have been introduced into Europe, little is known about the genetic diversity and relationships among these introduced germplasm resources. In Hungary, a living bamboo collection comprising 44 *Phyllostachys* species has been established and maintained for several decades [36,37]. However, the genetic diversity and relationships among these accessions have not yet been investigated. Therefore, the aim of the present study was to evaluate the genetic diversity and relationships among 96 genotypes representing 44 *Phyllostachys* species using eight simple sequence repeat (SSR) markers.

## 2. Results

### 2.1. Efficacy of Used SSR Primers

**Figure 1:**
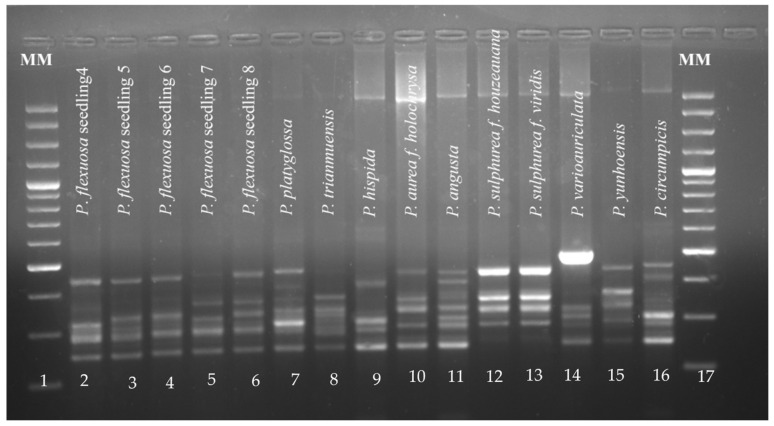
Agarose gel electrophoresis analysis of certain *Phyllostachys* genotypes. MM indicates the molecular size marker 100 bp DNA ladder (100–3000 bp).

**Figure 2:**
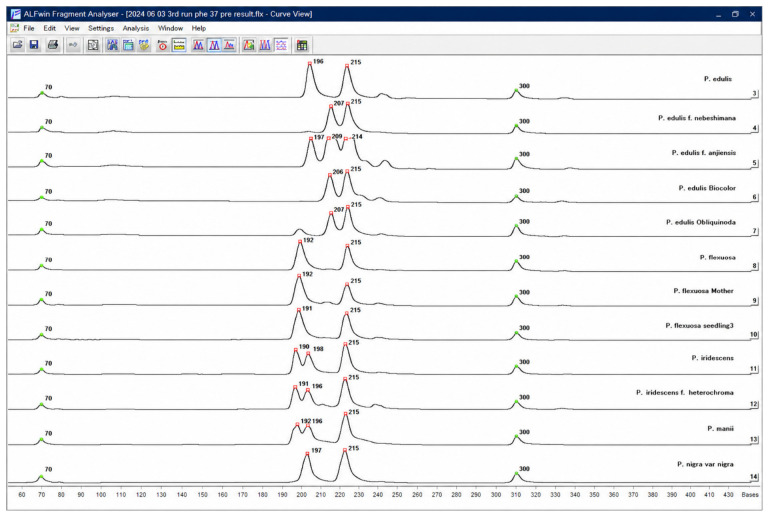
Polyacrylamide gel electrophoresis profile of PCR products amplified with the Phe37 SSR primer. Fragment sizes were determined using ALFwin Fragment Analysis Version 1.0 software (Amersham Pharmacia Biotech).

In the present study, simple sequence repeat analysis was conducted on 96 genotypes of the *Phyllostachys* genus to evaluate their genetic diversity using eight SSR markers. Following PCR amplification, amplicons were resolved by two electrophoresis methods: 2% agarose gel electrophoresis for products of the PBM021 primer (Figure 1) and 6% polyacrylamide gel electrophoresis for those of the other seven SSR primers (Figure 2).

**Figure 3:**
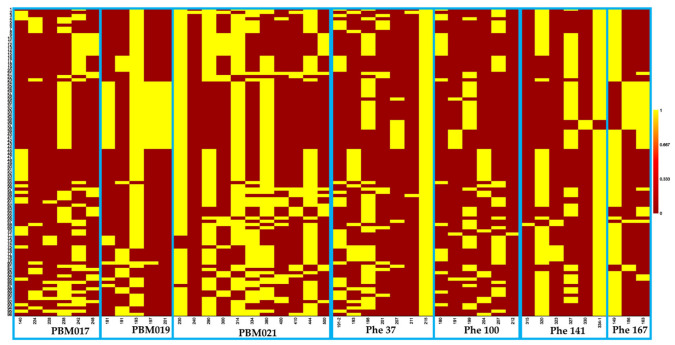
Allele distribution of SSR markers among 96 bamboo genotypes. Yellow represents alleles present, and maroon shows alleles absent. The sample numbers can be seen in Table 3.

The simple sequence repeat (SSR) markers used in this study exhibited considerable allelic variation, with 3–11 alleles per locus across the 96 *Phyllostachys* genotypes (Table 1), yielding a total of 47 alleles from the eight markers. Notably, PBM021 produced the highest number of 11 alleles, while Phe01 and Phe167 yielded the fewest (3 and 3). Seven of the eight SSR primers were polymorphic, with Phe01 being the sole monomorphic marker. Among the polymorphic markers, PBM021, PBM017, Phe100, and Phe37 showed elevated values across all diversity metrics (Table 1), whereas PBM019, Phe141, and Phe167 displayed comparatively lower values. Regarding the result of polymorphic information content (PIC), 6 markers were highly informative, as their PIC values exceeded 0.5, consistent with established criteria [38]. In contrast, Phe167 had its PIC value (0.48) less than 0.5. The distribution of alleles among the 96 genotypes is illustrated in Figure 3, where yellow denotes presence and maroon indicates absence.

Specifically, PBM017 amplified six alleles with frequencies ranging from 5.33% to 30.00%. PBM019 generated five alleles, among which the 193 bp fragment exhibited the highest frequency (48.85%); however, only the 201 bp allele was detected in *P. edulis* and its related forms, qualifying it as a private allele. PBM021 exhibited the greatest allelic richness (11), accompanied by a broad spectrum of frequencies from common to rare (240 bp, 410 bp, 500 bp and 300 bp at frequencies of 0.21%, 2.78%, 3.64% and 3.85%, respectively). Phe37 revealed seven alleles, with the 211 bp fragment being the least prevalent (0.86%) and the 215 bp fragment the most abundant (48.81%). Phe100 detected a single allele (212 bp) at 0.85% frequency, while Phe141 and Phe167 amplified alleles (334 bp and 149 bp) with maximum frequencies of 43.52% and 61.79%, respectively; Phe141 also identified a 315 bp allele at the lowest frequency of 0.46%. Overall, seven SSR markers displayed substantial variation in allele frequencies across the genotypes, with the exception of Phe01.

### 2.2. Genetic Variation in Bamboo Genotypes

**Figure 4:**
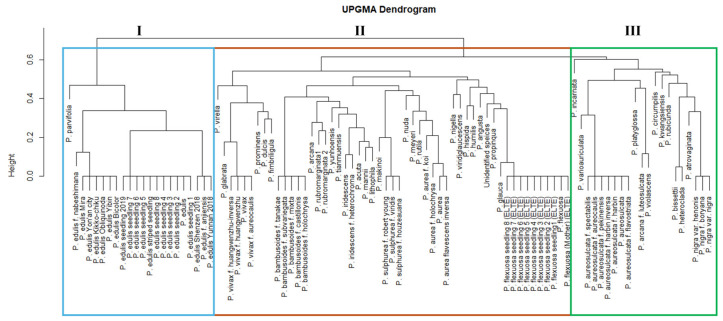
Cluster analysis of 96 bamboo genotypes according to 7 SSR markers, Cophenetic correlation: 0.893.

Figure 4 illustrates the genetic relationships among the 96 bamboo genotypes, as revealed by UPGMA clustering based on genetic distances. This analysis delineated three major clusters: Cluster I, comprising 22 genotypes of exclusively *P. edulis*, its close relatives, and *P. parvifolia* (indicating substantial genetic similarity between *P. parvifolia* and the *P. edulis* group, despite divergence from other species); the largest, Cluster II, with 54 genotypes spanning diverse species and forms (underscoring high genetic variation within this group); and Cluster III, encompassing the remaining 21 genotypes. Notably, the related forms *P. arcana* and *P. arcana f. luteosulcata* segregated into Clusters II and III, respectively—a pattern supported by prior research [35].

**Figure 5:**
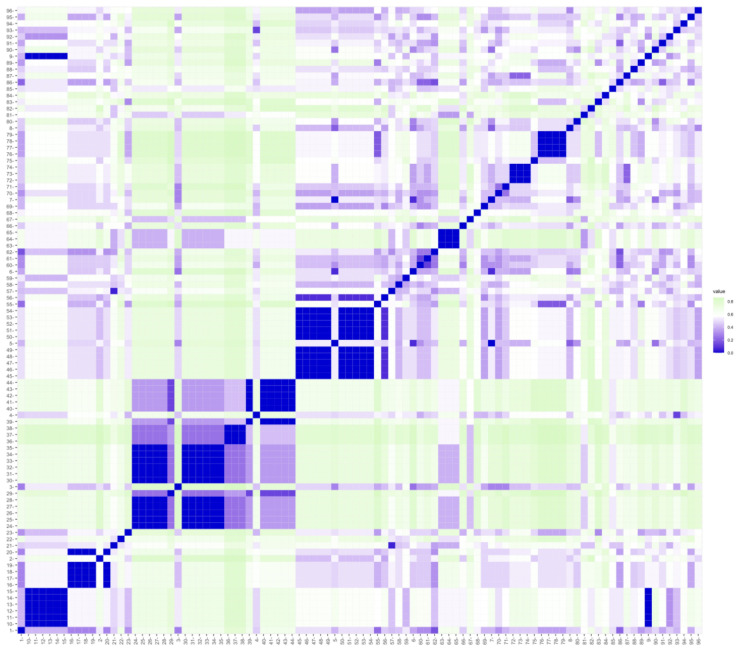
Heatmap of the genetic distance matrix for 96 bamboo genotypes. Genetic distance ranges from 0 (dark blue, no genetic distance) to 1 (green, highest genetic distance). The sample numbers can be seen in Table 3.

Figure 5 presents a heatmap of the genetic distance matrix for these 96 genotypes, using a color gradient from green (maximum distance) to blue (minimum). Genotypes in Cluster I (Figure 4) show pronounced distances from those in Clusters II and III, reinforcing the discrete groupings evident in the UPGMA dendrogram.

**Figure 6:**
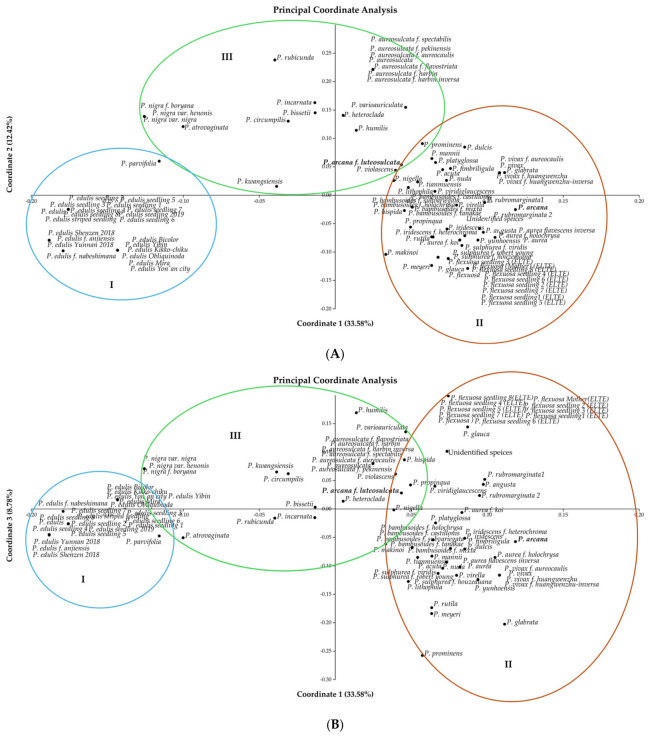
Principal Coordinate Analysis (PCoA) of 96 *Phyllostachys* bamboo genotypes based on Jaccard distances. (**A**) Plot of Coordinate 1 vs. Coordinate 2, explaining 46.00% of the total variation. (**B**) Plot of Coordinate 1 vs. Coordinate 3, explaining 54.78% of the total variation.

Principal coordinate analysis using Jaccard’s distances in PAST v4.17 further validated these clusters (Figure 6A,B). Table 2 summarizes the eigenvalues, percentages of variation explained, and cumulative percentages for the first ten principal coordinates, with the first two accounting for 45.84% (Coordinate 1 explaining 33.53% and Coordinate 2 explaining 12.31%) of total variation (82.11% by the tenth). The PCoA ordination confirms the UPGMA findings (Figure 4) through similar genotype groupings, bolstering confidence in the identified genetic clusters. Overlaps among certain genotypes, particularly *P. edulis* and its variants, indicate limited differentiation due to close relatedness or shared ancestral polymorphisms. Nonetheless, PCoA revealed nuanced intra-cluster dynamics: most genotypes formed compact clusters mirroring the UPGMA dendrogram, but some exhibited greater dispersion. For instance, *P. humilis*, *P. mannii*, *P. nigella*, and *P. prominens,* which were found in Cluster II in the UPGMA clustering dendrogram, aligned with Cluster III (green circle) in PCoA using Coordinates 1 and 2 (Figure 6A); similar variations appeared with Coordinates 1 and 3 (Figure 6B) for *P. humilis*, *P. nigella*, *and P. propinqua* (aligning with Cluster III) versus *P. platygossa* in Cluster II. PCoA also highlighted intra-specific differentiation, such as divergence of *P. vivax f. huangwenzhu* from *P. vivax*, *P. vivax f. aureocaulis*, *and P. vivax f. huangwenzhu-inversa*. Strikingly, green-encircled genotypes (Cluster III) showed broader dispersion despite overall cohesion, indicating higher diversity than suggested by UPGMA.

## 3. Discussion

In the present study, genetic variation was assessed among 96 bamboo genotypes, encompassing 44 *Phyllostachys* species, using eight SSR primer pairs. These markers proved highly informative, as indicated by high heterozygosity (H), polymorphism information content (PIC), Shannon index (H′), and Simpson diversity index (D) values (Table 1), indicating substantial polymorphism across the genotypes. PBM021 exhibited the highest PIC (0.847) and detected 11 alleles, indicating exceptional discriminatory ability among the studied genotypes. The allelic richness at loci indicates the high level of diversity [39]. The high allele richness in PBM021 suggests that this locus may describe substantial genetic variation within the germplasm of *Phyllostachys* taxa and could be potentially useful for genotype identification, germplasm management and conservation, and future breeding programs.

Allele frequency analysis is essential for evaluating genetic diversity, evolutionary dynamics, and adaptive potential in species [40]. The observed variation in allele frequencies across SSR markers underscored the genetic diversity among the 96 genotypes. In this study, we observed rare alleles in 4 primer sets; the 240 bp fragment (0.21%) at the PBM021 locus, the 211 bp allele (0.86%) at Phe37, the 212 bp allele (0.85%) at Phe100 and the 315 bp allele (0.46%) at Phe 141, all with frequencies below 1%, align with established definitions [41]. Although these alleles occur at low frequencies, they may represent unique components of genetic diversity. Their presence suggests that certain *Phyllostachys* taxa harbor uncommon genetic variation that contributes to the overall genetic resources of the germplasm collection, although their biological significance remains to be determined. Inversely higher-frequency alleles predominated in PBM019, Phe37, Phe141, and Phe167.

Notably, in this study PBM019 identified a private allele specific to *P. edulis* and its forms. Although a reference study reported the 201 bp allele in both *P. edulis* and *P. bambusoides* [34], it was exclusive to *P. edulis* and its forms in our analysis, absent from the five *P. bambusoides* forms examined.

The identification of a 201 bp private allele specific to *P. edulis* represents a significant finding with important potential applications. Private alleles can be used as diagnostic markers for germplasm identification and authentication, as they enable the unambiguous identification of a given species or form and help to detect mixed or mislabeled samples [42]. Due to these properties, they can also serve as “fingerprints” in germplasm collections, for example, for the verification of *Phyllostachys edulis*-derived material. In addition, they can be applied in breeding and selection, for example, in the verification of parental lines, confirmation of hybrids, and tracking of desirable genetic backgrounds, thereby forming a basis for marker-assisted selection. However, their applicability should be further validated using larger sample sizes and additional *Phyllostachys* species.

Our results align with prior investigations of SSR markers in *Phyllostachys* [34]. That study developed 30 polymorphic SSRs, including PBM017, PBM019, and PBM021, from *P. edulis (P. pubescens)* and evaluated polymorphism in *P. edulis* and six related species (including *P. atrovaginata*, *P. heteroclada*, *P. kwangsiensis*, and *P. bambusoides,* all represented here) [34]. They observed 100% polymorphism for PBM017 and PBM019 across those species [34]; our findings corroborate this pattern in the shared species. In contrast to their report of one allele per species at PBM021 in these four species [34], we identified 4–6 alleles per species; moreover, PBM021 amplified up to eight alleles in species such as *P. angusta*, *P. mannii*, *P. bissetii*, and *P. heteroclada*, all displaying heterozygosity across markers. Their analysis relied on silver-stained PAGE, whereas we employed Cy5-labeled forward primers with ALFexpress II Fragment Analyzer detection, offering superior sensitivity and quantification accuracy. Consequently, our data reveal greater PBM021 polymorphism in these *Phyllostachys* species than previously reported. As *Phyllostachys* species are allopolyploids [43,44], multiple alleles per locus are anticipated. Another study reported multiple alleles (ranging from 3 to 20) across 10 *Phyllostachys* species, with a maximum (17) from one SSR in one species [45]. Whole-genome duplication [46] and allopolyploidization [47] in *Phyllostachys* have generated homoeologous chromosomes [48], potentially explaining PBM021’s detection of up to eight alleles at a locus.

Jiang et al. similarly developed 20 polymorphic SSR markers, including Phe01, Phe37, Phe100, Phe141, and Phe167 (four of which were used in our study), for diversity and population structure analyses in *Phyllostachys*, focusing on *P. edulis* [28]. They applied these markers to 34 *P. edulis* populations in China, revealing moderate intraspecific diversity [28]. Whereas their work targeted *P. edulis* population structure, ours encompassed a wider spectrum of *Phyllostachys* species and forms. The pronounced polymorphism of shared markers (Phe37, Phe100, Phe141, and Phe167) here affirms their suitability for genus-wide studies. Within *P. edulis* and its forms, we detected no polymorphism in seedlings and minor differentiation among forms, contrasting Jiang et al.’s moderate diversity across populations [27,28]. This underscores the sampling strategy’s role in diversity assessment, with our results indicating stronger interspecific than intraspecific partitioning.

Assessing genetic diversity within and between bamboo populations is vital for conservation and breeding. Bamboo adaptation to fluctuating environments depends on its genetic reservoir. Analysis of 96 genotypes from 44 *Phyllostachys* species using seven polymorphic SSR markers revealed interspecific variation and limited intraspecific diversity. Cluster analysis of the 96 genotypes, based on the genetic distance matrix, delineated three clusters.

Cluster analysis identified three distinct *Phyllostachys* genotype groups. Cluster I primarily featured *P. edulis*, its forms, and *P. parvifolia*, reflecting their genetic proximity. This aligns with prior findings of similarity among *P. edulis* forms [30], despite reports of moderate [28] or low [49] diversity in Chinese/Japanese *P. edulis* populations via SSRs. Notably, while previous SSR analyses separated *P. edulis* and *P. parvifolia* [35,45], our clustering united them, likely due to marker and electrophoresis (PAGE) method differences.

Cluster II, the largest group, comprised 54 bamboo genotypes, including *P. propinqua* and *P. nuda*, consistent with previous reports [35]. Similarly, the placement of *P. rutila* and various forms of *P. bambusoides* in Cluster II aligns with those findings [35]. These three clusters corresponded to groupings observed among 10 *Phyllostachys* species using 54 EST-SSRs, where *P. glabrata*, *P. bambusoides*, and *P. aurea* were classified together [38]. Our results similarly positioned the diverse forms of *P. bambusoides*, *P. aurea*, and *P. glabrata* within the same cluster. Cluster III encompassed 21 genotypes, mainly seven forms of *P. aureosulcata*, *P. varioauriculata*, and *P. platyglossa*, corroborating prior observations [35]. Nuclear ribosomal DNA (nrDNA) internal transcribed spacer (ITS) sequences have been used to distinguish *Phyllostachys* species and related genera [50]. In that analysis, two main clusters emerged among *Phyllostachys* taxa; notably, *P. bissetii* and *P. heteroclada* co-occurred in one cluster, consistent with our results. Another AFLP-based study grouped *P. platyglossa*, *P. aureosulcata*, *P. bissetii*, *P. nigra*, and *P. heteroclada*, similar to our findings [35]. However, whereas *P. nigra*, *P. kwangsiensis*, and *P. sulphurea* clustered together [35], our analysis placed *P. nigra* and *P. kwangsiensis* in Cluster III and *P. sulphurea* in Cluster II. Furthermore, *P. incarnata* and *P. rubicunda* formed a distinct cluster from *P. platyglossa*, *P. aureosulcata*, *P. bissetii*, *P. nigra*, and *P. heteroclada* [50], whereas they co-clustered in our study. These inconsistencies likely arise from differences in SSR markers, polyacrylamide gel electrophoresis detection techniques, and clustering algorithms (neighbor-joining [50] versus UPGMA in this study). The recurrent clustering of *P. bissetii* and *P. heteroclada*, as well as *P. aureosulcata* and *P. platyglossa*, across studies underscores their close genetic relationships. In the present analysis, bamboo species and their related forms exhibited no genetic differentiation, as the SSR markers targeted identical genetic material within these variants. These observations are supported by prior reports indicating genetic similarity among related forms of *P. bambusoides*, *P. aureosulcata*, and *P. vivax* using SSR markers [30,51]. Comparative chloroplast genome analyses of eight *Phyllostachys* species, including forms of *P. nigra* and *P. vivax*, similarly revealed identity within *P. nigra* variants and among *P. vivax f. huangwenzhu* and *P. vivax f. aureocaulis*, despite morphological distinctions in leaf and culm coloration [51]. This consistency arises because SSR loci primarily target non-coding regions, which are unlikely to be tightly linked to color-regulating genes. Culm color variation in bamboo is chiefly governed by coding-region genes associated with pigment biosynthesis pathways and chloroplast functions [52,53].

Intriguingly, two related forms of *P. arcana* segregated into distinct clusters based on the three SSR markers used here, a pattern also noted previously [35]. In bamboos, morphological traits alone are often insufficient for reliable species identification because they are influenced by environmental conditions and phenotypic plasticity [54]. Furthermore, molecular studies have shown that reticulate evolution, including hybridization and introgression, can generate complex genetic patterns and obscure species boundaries [55,56]. Therefore, the observation that different forms of *P. arcana* were assigned to different clusters may reflect either the limitations of morphology-based taxonomy or historical gene flow among closely related taxa. Additional studies using broader sampling and more molecular markers are needed to determine whether these patterns represent intraspecific variation, cryptic speciation, or introgressive hybridization.

Kou et al. demonstrated that a carefully selected set of 24 SSR markers was sufficient to discriminate 20 forms of *Phyllostachys edulis* [57]. However, they also reported that the clustering of some taxa was not fully consistent with traditional taxonomy and suggested that this discrepancy might be related to the limited number of SSR markers used [57]. Therefore, it is possible that the use of only eight SSR markers in the present study limited the resolution of intraspecific relationships, including those observed among the different forms of *P. arcana*.

The PCoA plot (Figure 6A,B), derived from Jaccard distances, effectively illustrated genetic relationships among the 96 *Phyllostachys* genotypes. The first two principal coordinates explained 46.00% of the total variation, while Coordinates 1 and 3 accounted for 54.78%. Consistent with the dendrogram, PCoA delineated three clusters. Although the UPGMA dendrogram (Figure 4) provided a hierarchical depiction of relationships [58], PCoA offered a multidimensional representation of genetic distances and revealed the intermediate or overlapping patterns of genetic similarity. PCoA was largely consistent with UPGMA groupings but revealed patterns that were not apparent in the dendrogram. For example, *P. humilis,* which was in Cluster II by UPGMA, was placed closer to Cluster III in PCoA, whereas *P. platyglossa,* which was in Cluster III by UPGMA, was positioned among Cluster II genotypes in PCoA. These discrepancies likely occur from the methodological differences between the two approaches. The contrasting placements of these two species suggest that certain genetic relationships are represented differently by the two analytical methods. Nevertheless, the overall consistency between the UPGMA and PCoA analyses supports the robustness of the major clustering pattern identified among the *Phyllostachys* taxa.

Although the SSR markers effectively differentiated the bamboo genotypes in this study and revealed the genetic diversity, the present dataset comprising seven polymorphic loci should be regarded as an exploratory assessment of genetic relationships. Since the *Phyllostachys* genus has a complex allopolyploid nature, additional SSR or other molecular markers and genome-wide approaches are required to further resolve species-level relationships and taxonomic boundaries.

## 4. Materials and Methods

### 4.1. Plant Materials

Young, fully expanded leaves were collected from 96 bamboo accessions representing 44 *Phyllostachys* species. These accessions were primarily sourced from the Botanical Garden of the Hungarian University of Agriculture and Life Sciences in Gödöllő, Hungary, with additional samples obtained from Eötvös Loránd University in Budapest and locations in China. A comprehensive list of all genotypes is provided in Table 3.

### 4.2. Genomic DNA Extraction and PCR

Genomic DNA was extracted from the bamboo samples using the NucleoSpin^®^ Plant II kit (Macherey-Nagel GmbH & Co., Düren, Germany) according to the manufacturer’s instructions. DNA quality and quantity were evaluated using a Nanodrop 1000 Spectrophotometer (Thermo Fisher Scientific, Waltham, MA, USA) and 1% agarose gel electrophoresis. The extracted DNA was stored at −20 °C prior to use. Eight SSR primer pairs (Table 4), with the forward primer of each labeled using the fluorescent dye Cy5, were selected to assess genetic variation across the 96 bamboo genotypes. A touchdown PCR protocol was employed, which mitigates non-specific binding and primer dimer formation through an initial high annealing temperature, thereby improving specificity and sensitivity as the temperature declines [59].

Reactions were conducted in 10 μL volumes comprising 2 μL of genomic DNA (20 ng/μL), 4.25 μL sterile water, 1 μL of DreamTaq buffer (Thermo Fisher Scientific, Waltham, MA, USA) (10×), 1 μL of dNTPs (2 mM), 0.75 μL of forward and reverse primers (10 pM), 0.2 μL of MgCl_2_ (25 mM), and 0.05 μL of DreamTaq DNA polymerase (Thermo Fisher Scientific, Waltham, MA, USA) (5 U/μL).

The thermal cycling regime included initial denaturation at 95 °C for 2 min, followed by 10 cycles of 94 °C for 30 s, annealing decreasing from 65 °C to 55 °C by 1 °C per cycle for 30 s, and 72 °C extension for 1 min; this was followed by 24 cycles of 94 °C for 30 s, 56 °C for 30 s, and 72 °C for 1 min, concluding with a final extension at 72 °C for 5 min. All PCR were replicated three times per sample to verify reproducibility.

To estimate allele fragment sizes, PCR amplicons from seven SSR primers (excluding PBM021) were resolved using 6% polyacrylamide gel electrophoresis on an ALF express II Fragment Analyzer™ (Amersham Biosciences, Freiburg, Germany). Due to the larger fragment sizes produced by the PBM021 primer, its amplicons were analyzed separately using 2% agarose gel electrophoresis in 0.5× TBE buffer at 90 V for 1 h.

**Table 4:** Name and sequence of SSR primer used to evaluate genetic variation among tested bamboo samples. [34] *, [27] **.

No	Marker	5′ Sequence	Accession Number	Allele Sizes (bp) from the Reference *, **
1	PBM017	F-5′TATGCCTCCAATAATCCG R-3′AAGGCGTAGCCACCGAAT	FJ588776–FJ588781	245 (*P.atrovaginata*) *, 240 (*P. bambusoides*) *, 237 (*P. edulis*) *, 230, 242 (*P. heteroclada*) *
2	PBM019	F-5′TCCTAGCCCTACCCTGTCC3′ R-3′CACGGTGCTCGCTTAAATAG	FJ588789–FJ588794	191 (*P. atrovaginata*) *, 202 (*P. bambusoides*, *P. edulis*) *, 188 (*P. heteroclada*) *, 189 (*P. kwangsiensis*) *,
3	PBM021	F-5′AGGGTGTTATTTGCTATTGT R-3′AAATCCGACGCTGGAGGC	FJ588802–FJ588806	264 (*P. atrovaginata*) *, 279 (*P. bambusoides*) *, 260 (*P. edulis*) *, 301 (*P. heteroclada*) *
4	Phe01	F-5′CACCTCTTTCGTCATCAACC R-3′CGTAAACCCGGCAATCTA	FP093322	219–255 (*P. edulis*) **
5	Phe37	F-5′GCTCTTCGCCAAGTGCTAC R-3′ATCTTGTCCGTACCCAGGG	FP094642	196–213 (*P. edulis*) **
6	Phe100	F-5′GACATTAGGCGAGGTTCGG R-3′TCGTTTGGACAGGTAGAGGG	FP094809	189–204 (*P. edulis*) **
7	Phe141	F-5′AGGCCATAAGGAACTGCT R-3′CTACCCTCCAAACCTTCG	FP096517	321–336 (*P. edulis*) **
8	Phe167	F-5′AACAGCGAAACCACAGACC R-3′CCGAGCAGAGTAGGACGA	FP100624	151–163 (*P. edulis*) **

### 4.3. Data Analysis

Binary presence-absence data for alleles at each SSR locus were recorded across all 96 *Phyllostachys* genotypes. Several genetic diversity parameters were then computed for each SSR marker, including polymorphism information content (*PIC*), expected heterozygosity (*H*), Shannon’s diversity index (*H*′), and Simpson’s diversity index (D).

*PIC* was calculated using the formula

$$PIC=1-\sum_{i=1}^{n-1}{Pi}^{2}-\sum_{i=1}^{n}\sum_{j=1+1}^{n}2{Pi}^{2}{Pj}^{2}$$

where *n* is the number of alleles and *Pi* and *Pj* = allele frequencies in populations i and j, respectively [60].

Heterozygosity (*H*′) was calculated as $H=1-\;\sum_{i=1}^{l}{Pi}^{2}$, where *Pi* is the frequency of the ith allele across the total number of alleles l [61].

The Shannon index (*H*′), which measures overall diversity considering both allele number and frequency was measured with the following formula.

$H^{\prime }=\;-\sum \left(pi.\;In\;pi\right)$, where pi is the proportion of individuals in the ith species (ni/N), In is the Natural logarithm.

The Simpson diversity index (D), representing the probability that two randomly sampled individuals have different genotypes, was analyzed using the formula below.

$$D=1-\left(\frac{\Sigma ni\left(ni-1\right)}{N\left(N-1\right)}\right)$$

where *ni* is the number of individuals and *N* is the total number of species.

Genetic diversity among the 96 bamboo samples was assessed using the Unweighted Pair Group Method with Arithmetic Mean (UPGMA) clustering based on Jaccard’s similarity coefficient, calculated using the R program version 4.4.3. Principal Coordinate Analysis (PCoA), a method to visually represent genetic relationships in a two-dimensional plot, was performed using PAST 4.17 based on Jaccard’s distance.

## 5. Conclusions

This study evaluated the genetic diversity and genetic similarity relationships among 96 genotypes of *Phyllostachys* bamboo, encompassing 44 species, using eight SSR primer pairs. The markers exhibited substantial polymorphism, with the PBM021 primer showing the highest levels. This elevated polymorphism has significant implications for conservation and breeding programs, as it indicates an expansive gene pool with enhanced prospects for adaptation and selection. Indeed, the PBM021 marker proved particularly effective for differentiating interspecific variations in bamboo. *Phyllostachys* species have undergone whole-genome duplication and allopolyploidization, resulting in homoeologous chromosomes derived from diploid progenitors. Consequently, the PBM021 SSR locus likely amplifies products from these homoeologous chromosomes, amplifying up to eight alleles in certain species. Future studies should elucidate the underlying mutational mechanisms through cloning and sequencing of PBM021 amplicons to ascertain their genomic origins. Moreover, comprehensive bamboo genome analyses are needed to map SSR locus distributions and determine whether amplified alleles derive from homoeologous chromosomes or multiple non-coding regions.

Notably, the identification of a private allele underscores the presence of unique genetic variation within *P. edulis.* Clustering and PCoA analyses further confirm the genetic separation between *P. arcana* and *P. arcana f. luteosulcata*, consistent with prior reports; these distinctions call for high-resolution genomic studies to resolve these intrageneric and taxonomic relationships. Compared to previous studies limited to fewer *Phyllostachys* species, this work provides novel insights into genetic variability across a broader assemblage. Overall, the present SSR dataset provides preliminary molecular characterization and genetic diversity and genetic similarity evaluation among the tested *Phyllostachys* genotypes. While the identified clustering patterns contribute to germplasm characterization and management, the limited number of SSR loci does not provide sufficient definition of species-level relationships or taxonomic identification within this complex allopolyploid genus. Further studies with additional molecular markers, genome-wide approaches, and broader sampling are required to clarify evolutionary relationships within *Phyllostachys* taxa.

## Figures and Tables

**Table 1:** Information on SSR markers used in this study (OBL—number of observed alleles, PA—polymorphic allele, PPA—percentage of polymorphic allele (%), H—Heterozygosity, PIC—polymorphic information content, H′—Shannon Index, D—Simpson Diversity Index, OBZ—observed allele sizes and their distribution).

Locus Name	OBL	PA	PPA	H	PIC	H′	D	OBZ
PBM017	6	6	100	0.788	0.756	1.65	0.797	140 bp (16.67%), 224 bp (9.33%), 228 bp (5.33%), 238 bp (30.00%), 242 bp (25.33%), 248 bp (13.33%).
PBM019	5	5	100	0.695	0.663	1.40	0.712	181 bp (15.52%), 191 bp (10.92%), 193 bp (48.85%), 197 bp (12.64%), 201 bp (12.07%)
PBM021	11	11	100	0.863	0.847	2.11	0.867	230 bp (19.70%), 240 bp (0.21%), 290 bp (14.13%), 300 bp (3.85%), 314 bp (17.13%), 334 bp (8.57%), 380 bp (14.56%), 400 bp (2.78%), 410 bp (2.78%), 444 bp (12.63%), 500 bp (3.64%)
Phe 01	3	0	0	-	-	-	-	207 bp (100%) 219 bp (100%) 231 bp (100%)
Phe37	7	6	85.7	0.736	0.702	1.56	0.743	191 bp (13.36%), 193 bp (9.48%), 198 bp (23.71%), 201 bp (5.60%), 207 bp (5.17%), 211 bp (0.86%), 215 bp (41.81%).
Phe 100	6	6	100	0.769	0.735	1.56	0.779	180 bp (17.09%), 191 bp (7.69%), 199 bp (29.06%), 204 bp (16.24%), 207 bp (29.06%), 212 bp (0.85%).
Phe 141	6	6	100	0.675	0.615	1.25	0.685	315 bp (0.46%), 320 bp (28.70%), 323 bp (3.24%), 327 bp (22.69%), 330 bp (1.39%), 334 bp (43.52%).
Phe 167	3	3	100	0.542	0.480	0.92	0.560	149 bp (61.79%), 156 bp (14.63%), 163 bp (23.58%).

**Table 2:** Eigenvalues, percentage, and cumulative percentage of variation for 10 principal coordinates.

Coordinate	1	2	3	4	5	6	7	8	9	10
Eigenvalue	6.69	2.47	1.75	1.27	1.05	0.89	0.79	0.62	0.49	0.41
Percentage	33.58	12.42	8.78	6.38	5.28	4.45	3.96	3.10	2.47	2.04
Cumulative %	33.58	46.00	54.78	61.16	66.44	70.90	74.86	77.95	80.42	82.46

**Table 3:** List of *Phyllostachys* genotypes that were used in this study. ^1^—MATE, Gödöllő, Hungary. ^2^—ELTE, Budapest, Hungary. ^3^—China.

No.	Genotypes	No.	Genotypes
1	*P. acuta* ^1^	49	*P. flexuosa* seedling 3 ^2^
2	*P. angusta* ^1^	50	*P. flexuosa* seedling 4 ^2^
3	*P. arcana* ^1^	51	*P. flexuosa* seedling 5 ^2^
4	*P. arcana f. luteosulcata* ^1^	52	*P. flexuosa* seedling 6 ^2^
5	*P. aurea* ^1^	53	*P.* flexuosa seedling 7 ^2^
6	*P. aurea f. holochrysa* ^1^	54	*P. flexuosa* seedling 8 ^2^
7	*P. aurea f. flavescens inversa* ^1^	55	*P. glabrata* ^1^
8	*P. aurea f. koi* ^1^	56	*P. glauca* ^1^
9	*P. aureosulcata* ^1^	57	*P. heteroclada* ^1^
10	*P. aureosulcata f. flavostriata* ^1^	58	*P. hispida* ^1^
11	*P. aureosulcata f. harbin* ^1^	59	*P. humilis* ^1^
12	*P. aureosulcata f. harbin inversa* ^1^	60	*P. iridescens* ^1^
13	*P. aureosulcata f. pekinensis* ^1^	61	*P. iridescens f. heterochroma* ^1^
14	*P. aureosulcata f. aureocaulis* ^1^	62	*P. mannii* ^1^
15	*P. aureosulcata f. spectabilis* ^1^	63	*P. nigra f. boryana* ^1^
16	*P. bambusoides f. castillonis* ^1^	64	*P. nigra var. nigra* ^1^
17	*P. bambusoides f. holochrysa* ^1^	65	*P. nigra var. henonis* ^1^
18	*P. bambusoides f. mixta* ^1^	66	*P. nuda* ^1^
19	*P. bambusoides f. subvariegata* ^1^	67	*P. parvifolia* ^1^
20	*P. bambusoides f. tanakae* ^1^	68	*P. platyglossa* ^1^
21	*P. bissetii* ^1^	69	*P. propinqua* ^1^
22	*P. circumpilis* ^1^	70	*P. rubromarginata*1 ^1^
23	*P. dulcis* ^1^	71	*P. rubromarginata* 2 ^1^
24	*P. edulis* ^1^	72	*P. sulphurea f. viridis* ^1^
25	*P. edulis* seedling 1 ^1^	73	*P. sulphurea f. houzeauana* ^1^
26	*P. edulis* seedling 2 ^1^	74	*P. sulphurea f. robert young* ^1^
27	*P. edulis* seedling 3 ^1^	75	*P. virella* ^1^
28	*P. edulis* seedling 4 ^1^	76	*P. vivax* ^1^
29	*P. edulis f. nabeshimana* ^3^	77	*P. vivax f. aureocaulis* ^1^
30	*P. edulis* seedling 8 ^1^	78	*P. vivax f. huangwenzhu* ^1^
31	*P. edulis* striped seedling ^1^	79	*P. vivax f. huangwenzhu*-*inversa* ^1^
32	*P. edulis* seedling 5 ^1^	80	*P. meyeri* ^1^
33	*P. edulis* seedling 6 ^1^	81	*P. atrovaginata* ^1^
34	*P. edulis* seedling 7 ^1^	82	*P. rubicunda* ^1^
35	*P. edulis* seedling 2019 ^1^	83	*P. fimbriligula* ^1^
36	*P. edulis* f. anjiensis ^1^	84	*P. incarnata* ^1^
37	*P. edulis* Yunnan 2018	85	*P. kwangsiensis* ^1^
38	*P. edulis* Shenzen 2018	86	*P. lithophila* ^1^
39	*P. edulis* Yibin ^3^	87	*P. makinoi* ^1^
40	*P. edulis f. bicolor* ^3^	88	*P. nigella* ^1^
41	*P. edulis f. obliquinoda* ^3^	89	*P. prominens* ^1^
42	*P. edulis f. heterocycla* ^3^	90	*P. rutila* ^1^
43	*P. edulis* Yon’an city ^3^	91	*P. tianmuensis* ^1^
44	*P. edulis* ‘Mira’ ^3^	92	*P. varioauriculata* ^1^
45	*P. flexuosa* ^1^	93	*P. violascens* ^1^
46	*P. flexuosa* (Mother) ^2^	94	*P. viridiglaucescens* ^1^
47	*P. flexuosa* seedling1 ^2^	95	*P. yunhoensis* ^1^
48	*P. flexuosa* seedling 2 ^2^	96	Unidentified ^1^

## Data Availability

The data presented in this study are available upon request from the corresponding author. The data are not publicly available due to privacy restriction.
